# Vagus nerve stimulation: mechanisms and factors involved in memory enhancement

**DOI:** 10.3389/fnhum.2023.1152064

**Published:** 2023-06-29

**Authors:** Laura K. Olsen, Ernesto Solis, Lindsey K. McIntire, Candice N. Hatcher-Solis

**Affiliations:** ^1^Air Force Research Laboratory, 711th Human Performance Wing, Cognitive Neuroscience, Wright-Patterson Air Force Base, OH, United States; ^2^Oak Ridge Institute for Science and Education, Oak Ridge, TN, United States; ^3^Air Force Research Laboratory, 711th Human Performance Wing, Aerospace Physiology, Wright-Patterson Air Force Base, OH, United States; ^4^Consortium of Universities of the Washington Metropolitan Area, Washington, DC, United States; ^5^Infoscitex Corporation, Dayton, OH, United States

**Keywords:** vagal nerve, peripheral nerve stimulation, locus coeruleus, synaptic plasticity, hippocampus

## Abstract

Vagus nerve stimulation (VNS) has been recognized as a useful neuromodulation tool to target the central nervous system by electrical stimulation of peripheral nerves. Activation of the nucleus of the solitary tract (NTS) in the brainstem by vagal afferent nerve fibers allows for modulation of various higher order brain regions, including limbic and cerebral cortex structures. Along with neurological and psychiatric indications, clinical and preclinical studies suggest that VNS can improve memory. While the underlying mechanisms to improve memory with VNS involve brain areas, such as the prefrontal cortex and processes including alertness and arousal, here we focus on VNS-induced memory improvements related to the hippocampus, the main area implicated in memory acquisition. In addition, we detail research demonstrating that a targeted approach to VNS can modify memory outcomes and delve into the molecular mechanisms associated with these changes. These findings indicate that a greater understanding of VNS mechanisms while also considering stimulation parameters, administration site, timing in relation to training, and sex-specific factors, may allow for optimal VNS application to enhance memory.

## 1. Introduction

In the late 1800s, the American neurologist James L. Corning initially used electrical stimulation of the vagus nerve, or vagus nerve stimulation (VNS), to treat epilepsy (Yuan and Silberstein, 2016). While Corning was unsuccessful, his pioneering work was instrumental for the inception of VNS research (Lanska, 2002; Yuan and Silberstein, 2016). Bailey and Bremer (1938) vagotomized cats to study the function of the vagus nerve and Zanchetti et al. (1952) found that VNS on cats caused global cortical desynchronization (Yuan and Silberstein, 2016). Nearly 100 years after Corning’s initial VNS research, Zabara used a strychnine-induced canine model of epilepsy to demonstrate that VNS could inhibit seizures (Zabara, 1992; Yuan and Silberstein, 2016). Shortly thereafter, clinical human studies of VNS as a therapeutic intervention to treat epilepsy were performed, and in 1997 the United States Food and Drug Administration (FDA) approved VNS as an implantable, electric pulse generator with a bipolar electrode cuff to treat refractory epilepsy (Schachter, 2002). Interestingly, in addition to modulating cortical excitability, VNS was found to affect mood, and in 2005 VNS was approved by the FDA as a treatment for drug-resistant depression (Berry et al., 2013). FDA approval of VNS for refractory epilepsy and depression paved the way for future studies examining the cognitive-enhancing effects of VNS.

Research exploring the cognitive-enhancing effects of invasive VNS used as an antiepileptic or antidepressant therapy found that VNS improved attention, arousal, short-term memory, verbal memory recognition, working memory, memory consolidation, mood, and decision-making in patients (Clark et al., 1999; Sackeim et al., 2001; Martin et al., 2004; Ghacibeh et al., 2006; Klinkenberg et al., 2013; Vonck et al., 2014; Sun et al., 2017; Broncel et al., 2022). However, some clinical studies reported inconsistent effects of VNS on memory (Dodrill and Morris, 2001; Helmstaedter et al., 2001; Sackeim et al., 2001; Klinkenberg et al., 2012). Several studies have explored the memory-enhancing effects of VNS used as an antiepileptic or antidepressant therapy, but few studies have investigated the effects of non-invasive VNS on memory in healthy humans. Non-invasive VNS administered to healthy adults has been shown to enhance creativity, alertness, and associative memory (Jacobs et al., 2015; Steenbergen et al., 2015; Colzato et al., 2018; Klaming et al., 2022). Similar to human trials, VNS augments learning, memory, and retention performance in rats (Clark et al., 1998; Sanders et al., 2019; Olsen et al., 2022). Harnessing the potential of VNS to enhance memory, including during times of memory impairment, requires a better understanding of the underlying mechanisms of VNS and the factors that modulate its effects.

Vagus nerve stimulation affects excitability in memory-associated pathways by altering brain neurotransmitters, such as γ-aminobutyric acid (GABA) and glutamate as well as the neuromodulators serotonin, dopamine and norepinephrine (Ben-Menachem et al., 1995; Krahl et al., 1998; Walker et al., 1999; Dorr and Debonnel, 2006; Manta et al., 2009, 2013). These alterations lead to lasting functional changes at synapses facilitating transmission and strengthening processes underlying memory. Modulation of these systems *via* VNS may allow for task-specific performance improvement or sustained basal level function during times of cognitive decline. Many reviews have explored the interaction of VNS and memory, but the mechanisms underlying VNS-induced effects on memory and the experimental parameters influencing these effects remain unclear (Boon et al., 2006; Vonck et al., 2014). Here, we review the mechanisms of VNS-induced memory enhancement and go over factors that should be considered in a targeted approach to VNS, including administration site, timing in relation to learning, and sex-specific differences.

## 2. Mechanisms of VNS-induced memory enhancement

**FIGURE 1:**
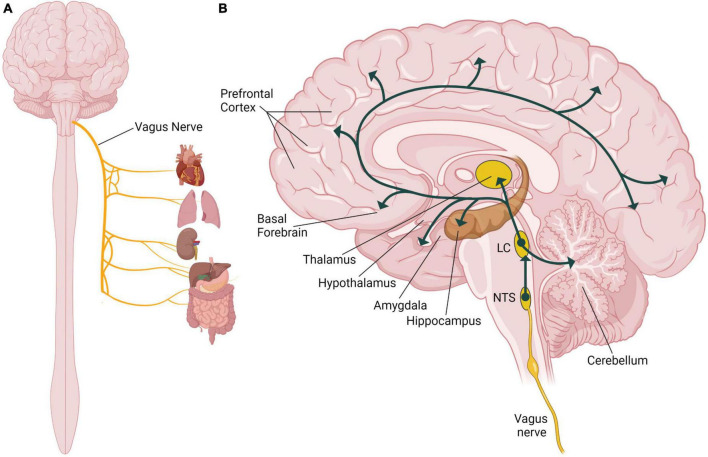
Role of the vagus nerve in the human nervous system. **(A)** Illustration of the organs innervated by the vagus nerve including the heart, lungs, kidneys, and the digestive tract. **(B)** Illustration of a sagittal section of the human brain showing the vagal afferent pathway through the nucleus of the solitary tract (NTS), the locus coeruleus (LC) and different brain areas including components of the limbic system, cortex, and the cerebellum. Illustration focuses on the vagal nerve projections to areas of the brain that are associated with known effects of vagus nerve stimulation (VNS) on memory. Created with BioRender.com.

The vagus nerve, also known as the tenth cranial nerve (or CN X), is a part of the autonomic nervous system regulating many involuntary body functions to maintain homeostasis (Howland, 2014). The vagus nerve is the longest cranial nerve extending from the brain through the thorax to the abdomen (Berthoud and Neuhuber, 2000), and as the primary nerve in the parasympathetic nervous system it regulates blood pressure, heart rate, respiration, digestion, and the immune response (Figure 1A; Agostoni et al., 1957). The vagus nerve is composed of approximately eighty percent sensory afferent nerve fibers and twenty percent motor efferent fibers, and it carries sensory information from peripheral organs to the brain and sends motor signals from the brain to those organs relaying signals bidirectionally (Broncel et al., 2020). Afferent sensory vagus nerve fibers primarily project to the nucleus of the solitary tract (NTS) in the brainstem (Figure 1B). The NTS is the main integration center for the vagal sensory afferent pathways (Cooper et al., 2021). The sensory afferent fibers then project from the NTS to the main noradrenergic center of the brain, the locus coeruleus. Neurons from the locus coeruleus project to many different areas of the brain, including areas implicated in processes associated with memory (Szabadi, 2013; Bari et al., 2020; Figure 1B). The locus coeruleus innervates the amygdala and the hippocampus, areas of the brain that are important for emotional and episodic memories (Sara and Devauges, 1988; Berthoud and Neuhuber, 2000; Takeuchi et al., 2016). The thalamus and hypothalamus also receive noradrenergic input from the locus coeruleus and are involved in sensory processing, wakefulness and the stress response (Figure 1B; Beas et al., 2018; Rodenkirch et al., 2019). The basal forebrain, a region critical for regulating alertness and arousal, is innervated by the locus coeruleus (Figure 1B; Schwarz and Luo, 2015; McBurney-Lin et al., 2019). The prefrontal cortex, which is essential for regulating attention and higher order cognitive processes like decision-making, also receives noradrenergic stimulation from the locus coeruleus (Figure 1B; Schwarz and Luo, 2015; McBurney-Lin et al., 2019; Bari et al., 2020). The locus coeruleus also innervates the midbrain and the dorsal raphe nucleus to respectively influence dopaminergic and serotonergic signaling in the brain, which can have effects to modulate many behaviors (Bari et al., 2020). Through its physiological connections to multiple brain areas, the vagus nerve is able to modulate many behavioral processes involved in memory.

**FIGURE 2:**
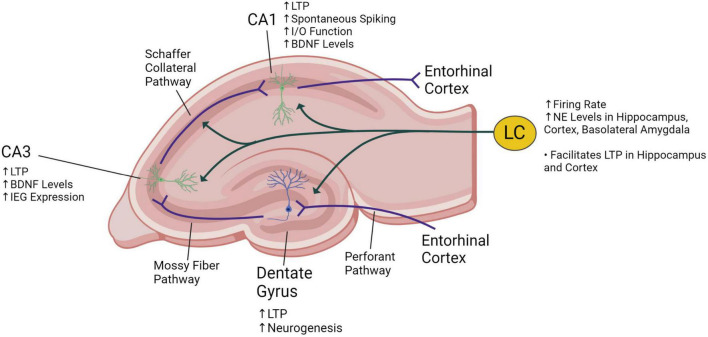
Illustration of a coronal section of the rat hippocampus showing innervation of the locus coeruleus (LC) into the dentate gyrus, CA1 and CA3 areas. The trisynaptic circuit consists of the three synaptic connections made at the dentate gyrus (from entorhinal cortex fibers entering the hippocampus), at the CA3 (from mossy fibers stemming from neurons at the dentate gyrus) and at the CA1 (from Schaffer collateral fibers stemming from neurons at the CA3). The known effects of vagus nerve stimulation on the locus coeruleus and the hippocampus are described. VNS increases norepinephrine (NE) levels, long-term potentiation (LTP), brain-derived neurotrophic factor (BDNF) expression, immediate early gene (IEG) expression, and the input/output (I/O) function. Created with BioRender.com.

Electrical stimulation of the vagus nerve modulates activity in the dorsal motor nucleus of the vagal nerve, nucleus ambiguus, NTS, and the trigeminal nerve (Baker and Lui, 2023), resulting in various physiological responses due to downstream effects of these targets (Aston-Jones et al., 1980). The NTS receives the majority of vagal afferent synapses between these nuclei and is activated by VNS (Cooper et al., 2021). Excitatory projections from the NTS can activate locus coeruleus neurons promoting the release of noradrenaline throughout the brain in the limbic structures, cerebral cortex, and cerebellum (Figure 1B; Bari et al., 2020). In a preclinical model, acute VNS was found to increase the firing rate of norepinephrine neurons in the locus coeruleus (Dorr and Debonnel, 2006). Additional animal studies indicate VNS increases the concentration of norepinephrine in the cortex, hippocampus, amygdala, and cerebral spinal fluid (Hassert et al., 2004; Roosevelt et al., 2006; Follesa et al., 2007; Shen et al., 2012). Preclinical investigations of the locus coeruleus and hippocampus have found the neurological consequences of VNS to be dependent on stimulation parameters (Clark et al., 1998; Groves and Brown, 2005). In rats, VNS increases the concentration of norepinephrine in the hippocampus and cortex in an intensity-dependent manner (Roosevelt et al., 2006). Based on preclinical studies, VNS activates afferent fibers increasing the neuronal firing rate in the locus coeruleus resulting in a higher norepinephrine concentration in brain regions implicated in memory formation (Figure 2).

It is known that arousal states are regulated by locus coeruleus firing and subsequent norepinephrine release in the basal forebrain (Joshi et al., 2016; Collins et al., 2021; Sharon et al., 2021). Accordingly, in a preclinical study VNS increased the arousal state in mice, characterized by widespread excitatory activation of cortical subregions, which was coupled and partially attributed to the activation of the noradrenergic system (Collins et al., 2021). Although the mechanisms are poorly understood, arousal modulates memory formation and retention (Sharot and Phelps, 2004; Clark-Foos and Marsh, 2008; Mneimne et al., 2010). In a clinical study where VNS improved working memory in patients with epilepsy, electroencephalogram recordings were obtained and it was found that VNS produces brain activity that resembles the brain responses observed for improved levels of attention (Sun et al., 2017). This supports that higher arousal levels and attentional mechanisms play a role in VNS-induced memory enhancement. While these findings suggest that VNS-induced memory improvements involve cortical regions modulating arousal, in the rest of the review we focus on VNS-induced memory enhancements related to increased norepinephrine levels in the hippocampus.

Most of the evidence that VNS affects activity in the locus coeruleus and subsequently synaptic plasticity in the hippocampus is from preclinical animal studies. As shown in Figure 2, the trisynaptic circuit (or trisynaptic loop) is implicated in synaptic plasticity and is a relay of information starting with signals entering the hippocampus from the entorhinal cortex through the perforant pathway. Initially, perforant pathway fibers synapse onto granule cells located at the dentate gyrus. Then, granule cells send projections through the mossy fiber pathway to pyramidal neurons at the CA3, and these neurons send signals through the Schaffer collateral pathway to other pyramidal neurons in the CA1. Finally, information exits the hippocampus back to the entorhinal cortex, completing the trisynaptic loop (Doller and Weight, 1982; Herreras et al., 1987). VNS may enhance hippocampal synaptic plasticity through locus coeruleus-mediated adrenergic signaling affecting the trisynaptic circuit (Vonck et al., 2014). Activation of the locus coeruleus can induce β-adrenergic receptor-dependent potentiation of perforant path-dentate gyrus evoked potentials in granule cells (Walling and Harley, 2004). VNS can enhance perforant path-CA3 field excitatory post-synaptic potentials that are dependent on locus coeruleus-mediated β-adrenergic receptor signaling (Shen et al., 2012; Figure 2). Long-term potentiation (LTP), characterized by enhanced postsynaptic responses after stimulation, is a form of hippocampal synaptic plasticity that is associated with synaptic strengthening and increased learning and memory (Havekes et al., 2011; Kandel et al., 2014). In rats, it has been shown that VNS increases LTP in the dentate gyrus (Zuo et al., 2007) and the CA1 (Olsen et al., 2022) subregions of the hippocampus (Figure 2). Additionally, in the CA1 region of the hippocampus, VNS increases excitability or spontaneous spiking and enhances synaptic transmission as measured by the input/output (I/O) function (Olsen et al., 2022; Figure 2).

VNS also induces lasting changes in synaptic plasticity by altering mediators of synaptic plasticity, such as growth factors, LTP, and immediate early genes (IEGs). In the CA1 and CA3 subregions of the hippocampus, VNS increases the expression of brain-derived neurotrophic factor (BDNF), a neurotrophin that promotes plasticity, neuronal survival, and neurogenesis (Figure 2; Biggio et al., 2009; Olsen et al., 2022). VNS also activates the phosphorylation and enhances expression of tropomyosin receptor kinase B (TrkB), the receptor for BDNF (Furmaga et al., 2012). Similarly, in the hippocampus VNS increases fibroblast growth factor expression, which modulates cell proliferation (Follesa et al., 2007). Expression of the IEGs *arc* and *cfos* in the cortex are also enhanced by VNS (Sanders et al., 2019). IEGs play an essential role in learning and long-term neural plasticity (Perez-Cadahia et al., 2011). VNS also increases the expression of adrenergic receptors, which facilitate LTP in the hippocampus (Shen et al., 2012). Expression of proteins critical for LTP including GluN2B, a subunit of the N-methyl-D-aspartate (NMDA) receptor, and its downstream signaling target calcium/calmodulin-dependent protein kinase II (CaMKII) is also increased by VNS (Alvarez-Dieppa et al., 2016). Overall, VNS induces long-term changes in plasticity and memory consolidation by increasing the expression of growth factors, IEGs, and LTP proteins in the hippocampus.

## 3. Factors influencing VNS-induced memory enhancement

Studies performed over the last several decades indicate that VNS has the potential to modify memory in humans and rodents (Clark et al., 1998; Sanders et al., 2019; Olsen et al., 2022). Here, we summarize clinical studies specifically focused on memory-based tasks, such as recognition and working memory that are likely affected by VNS-induced changes in synaptic plasticity (see Table 1). These clinical studies have shown inconsistent VNS-induced memory improvements, indicating further optimization of VNS application is warranted to maximize the potential memory-enhancing benefits of VNS. This section reviews the experimental design and results of VNS studies focused on memory and provides evidence that a targeted approach to VNS application, in which additional factors are considered, may maximize memory performance benefits.

**TABLE 1:** Summary of vagus nerve stimulation (VNS) in humans comparing the effects of cervical and auricular stimulation.

References	Type of VNS	Task	Timing of VNS	Male (M)/Female (F) ratio (shown as %)		Subject condition	Effect of VNS
				SHAM (%)	VNS (%)		
Klaming et al., 2022	tcVNS	Recognition	Before learning	53M/47F	73M/27F	Healthy adult	Fewer false negative errors in recognition task (*p* < 0.05)
Sun et al., 2017	dcVNS	Working memory	Before/during learning	N/A	60M/40F	Epileptic adult patients	Fewer errors in working memory task (OR = 0.63)
Clark et al., 1999	dcVNS	Recognition	After learning	N/A	Not reported	Epileptic adult patients	Improved memory retention in verbal memory task (*p* < 0.05)
Hoppe et al., 2001	dcVNS	Recognition	Random	N/A	72M/27F	Epileptic adult patients	No effect on verbal/figural learning tasks (*p* > 0.05)
McGlone et al., 2008	dcVNS	Memory Observation Questionnaire	Random	N/A	56M/44F	Epileptic adult patients	No effect on objective memory scores/memory complaints (*p* > 0.05)
Ghacibeh et al., 2006	dcVNS	Recall/Recognition	After learning or before recall/recognition	N/A	50M/50F	Epileptic adult patients	VNS after learning improved retention in verbal memory task (*p* < 0.01)
McIntire et al., 2021	tcVNS	Multi-tasking	Before/after task	85M/15F	80M/20F	Healthy sleep fatigued adults	Less fatigued induced decline (*p* < 0.05)
Mertens et al., 2022	dcVNS and taVNS	Recognition	After learning	N/A	33M/67F	Epileptic adult patients	No acute effect on word recognition task (*p* > 0.05)
Mertens et al., 2020	taVNS	Recognition	After learning	N/A	Younger = 49M/51F Older = 29M/70F	Healthy adults	No effect on recall or recognition (*p* > 0.05)
Jacobs et al., 2015	taVNS	Associative memory	During/after learning	N/A	50M/50F	Older adults	Higher correct hits in face-name task (*p* < 0.05)
Helmstaedter et al., 2001	dcVNS	Recognition	During testing	N/A	Not reported	Epileptic adult patients	Reversible decline in figural recognition (*p* < 0.05)

Experimental design parameters in clinical vagus nerve stimulation (VNS) studies investigating memory performance. Application of VNS in clinical studies was found to have varied success in improving memory in specific behavioral tasks. Experimental factors, including site of VNS application, timing of VNS administration in relation to task, and sex, may mediate efficacy of VNS-induced memory enhancement. Where available the proportion of male to female for the human subjects participating in the studies was expressed as a% [under male (M)/female (F) ratio]. dcVNS, direct cervical vagus nerve stimulation; taVNS, transcutaneous auricular vagus nerve stimulation.

### 3.1. Stimulation parameters of VNS

Inconsistencies in VNS-induced memory improvements have been attributed to differences in stimulation parameters used in VNS studies, such as intensity, frequency, and duration (Clark et al., 1999, Aniwattanapong et al., 2022). However, VNS stimulation parameters are dependent on the study, the individual subject, and the type and location of VNS application. Most of the clinical studies presented in Table 1 utilize cervical VNS (dcVNS), which requires stimulation intensities ranging from 0.5–2.75 mA and repeated stimulation (30 Hz, 30 s duration). Transcutaneous cervical VNS (tcVNS) uses a VNS device to non-invasively stimulate at different intensities depending on the subject to account for individual differences in muscle activation threshold. Since tcVNS stimulates the vagus nerve through the skin at the neck, the intensity required can be as high as 60 mA (25 Hz, 2 min duration at each side of the neck for a total stimulation of 4 min). For transcutaneous auricular VNS (taVNS), the 2 instances with no positive outcome applied 0.1 mA or 0.33–0.69 mA with both stimulating for 30 s at 25 Hz (Mertens, 2020, 2022), and the study showing a memory improvement stimulated with 5.0 mA for 17 min at 8 Hz (Jacobs et al., 2015). However, the intensity for taVNS can be as high as 50 mA and optimization of stimulation parameters is warranted. Thus, the type of VNS used requires specific stimulation parameters. Further discussion about the influence of VNS parameters, including intensity, frequency and waveform, on therapeutic outcomes has been reviewed previously (Yap et al., 2020, Aniwattanapong et al., 2022). While stimulation parameters when applying VNS is an important factor contributing to its effectiveness, experimental design parameters have seldom been examined as a source for variation in VNS-induced phenotypes.

### 3.2. Anatomical location of VNS

Recently, research into electroceuticals as cognitive enhancers in healthy humans has expanded. In particular, transcutaneous VNS (tVNS) has gained popularity because it is non-invasive, user-friendly and well-tolerated. The rise in companies producing commercial off-the-shelf wellness versions of tVNS, for both tcVNS and taVNS, is further evidence of its growing popularity. Interestingly, application of VNS at the cervical or auricular site may have different behavioral effects. As described in Table 1, VNS applied to the cervical branch of the vagus nerve appears to more consistently improve performance in multi-tasking/recognition memory tasks than when applied to the auricular site (Clark et al., 1999; Sun et al., 2017; McIntire et al., 2021; Klaming et al., 2022). An explanation for the discrepancy in VNS effectiveness to modulate memory between cervical and auricular sites is the anatomical differences of their respective vagus nerve branches, such as number of myelinated fibers (Safi et al., 2016). Although clinical studies suggest tcVNS produces more consistent results, taVNS is often preferable over tcVNS because of its user-friendly hardware design. Although evidence suggests that taVNS is capable of improving memory performance (Jacobs et al., 2015), more studies are warranted to optimize taVNS and produce consistent cognitive enhancements.

### 3.3. Relative timing of VNS

As detailed in Table 1, some VNS studies have taken a targeted approach to VNS administration by pairing VNS with a specific learning or performance task (Ghacibeh et al., 2006; Sun et al., 2017; McIntire et al., 2021; Klaming et al., 2022), whereas other studies applied a non-targeted approach by exploring the effects of general VNS (Hoppe et al., 2001; McGlone et al., 2008; Klinkenberg et al., 2013). Preclinical studies suggest that timing VNS around memory consolidation is key to modulating memory and effectiveness of learning (Clark et al., 1995, 1998; Sanders et al., 2019). In a preclinical model, VNS enhanced recognition memory performance and increased expression of hippocampal BDNF mRNA when the timing of stimulation overlapped with memory consolidation, whereas BDNF mRNA expression did not increase when VNS was not paired with a training task (Sanders et al., 2019). VNS-induced increases in LTP/synaptic plasticity protein expression coinciding with memory acquisition and consolidation may explain the sensitivity of VNS timing in clinical studies. For example, applying VNS during the testing phase was unable to modulate performance because stimulation was paired with memory-retrieval instead of encoding and consolidation (Helmstaedter et al., 2001). Further research will help elucidate the relationship between VNS timing and memory performance.

### 3.4. Sex-specific differences of VNS

To date, no clinical study has investigated sex-specific differences regarding the effectiveness of VNS to improve cognition. However, a review of the clinical VNS literature and reported male/female ratios suggests that there may be sex-specific differences (see Table 1). Several studies pairing VNS with a learning paradigm and showing an enhancement in memory reported an equal ratio of male versus females or disproportionately favored males (Ghacibeh et al., 2006; Sun et al., 2017; McIntire et al., 2021; Klaming et al., 2022). However, other studies disproportionately favoring females found no significant influence of VNS on performance despite using a targeted approach to VNS timing (Mertens et al., 2020, 2022). The disparity between male/female representation among study participants and inconsistent significant outcomes suggest that the memory-enhancing effects of VNS may be modulated by sex. With this possibility in mind, sex is discussed in this review as an experimental parameter that may require further optimization for VNS to maximally improve memory for both males and females.

Although no study has explicitly investigated the influence of sex on VNS-induced memory enhancement, sex-specific differences in response to VNS are known for other outcomes. Females present with higher levels of vagal parasympathetic activity when compared to men (Koenig and Thayer, 2016). Recent studies have indicated that sex mediates the cardiovascular and parasympathetic response to VNS in rodents and humans (De Couck et al., 2017; Yaghouby et al., 2020; Yokota et al., 2022). Additionally, chronic VNS significantly changes circadian rhythms in rats in a sex-specific manner (Groff et al., 2020). In humans, taVNS reduces heat pain perception in men, but not women (Janner et al., 2018). With sex modulating multiple effects of VNS, it is necessary to investigate the influence of sex on VNS-induced memory enhancement.

Currently, there is limited research available to explain the sex-specific differences in VNS-mediated memory improvements. Anatomical human cadaver investigation of male and female cervical vagus nerve found no sex-specific differences in fascicle nerve number or vascularity of the vagus nerve (Hammer et al., 2018). A rodent study that paired VNS with motor training found VNS to induce reorganization of cortical motor maps among males and females (Tseng et al., 2020). This study further validates the ability of VNS to promote synaptic plasticity when a targeted approach to timing is utilized (Tseng et al., 2020). Further research is warranted to determine if sex-specific differences in plasticity underlie the inconsistent effects of VNS on memory.

## 4. Discussion

Since the 1990s, researchers have been investigating VNS as a tool to modulate memory. Although the literature has established the ability of VNS to enhance memory, inconsistent findings suggest the need for a better understanding of this stimulation treatment and the underlying mechanisms. Recent preclinical studies have identified hippocampal synaptic plasticity pathways that may contribute to VNS-induced memory enhancement. These findings lay the groundwork for a better understanding of VNS at the mechanistic level, but many questions remain unanswered. As outlined in this review, the efficacy of VNS as a memory-enhancing application appears to be dependent upon experimental parameters. Using a targeted approach by pairing VNS with learning appears to be essential for successful strengthening of memory formation. This targeted VNS strategy may increase positive memory performance outcomes presumably because VNS further increases synaptic plasticity during the learning process. The site of VNS application and sex of the individual receiving VNS may also mediate the effectiveness of VNS to improve memory. However, sex and site-specific differences in VNS outcomes require further mechanistic investigation. VNS is a promising strategy for neuromodulation, and collaboration between clinical and preclinical investigations will identify the optimal approach for VNS-induced memory enhancement.

## Author contributions

LO, ES, LM, and CH-S: literature review and manuscript preparation. ES and CH-S: figures preparation. All authors contributed to the article and approved the submitted version.
